# Implementation of Guidelines and a Decision Support Tool for Blood Glucose Monitoring Device Prescriptions in Saudi Arabia: Compliance Assessment and Potential Cost Savings and Coverage Expansion

**DOI:** 10.7759/cureus.97804

**Published:** 2025-11-25

**Authors:** Lamya Alzubaidi, Faisal Elenezi, Amera S Alrshood, Abdullah Alsoheimi, Abdullah M Arabe, Muna Hassan Mustafa Hassanein

**Affiliations:** 1 Ada'a Health Center, Ministry of Health, Riyadh, SAU; 2 Hospitals Services Assistant Agency, Ministry of Health, Riyadh, SAU; 3 General Directorate of Specialized Centers Affairs, Ministry of Health, Riyadh, SAU; 4 Training and Institutional Development Department, Branch of the Ministry of Health, Riyadh, SAU

**Keywords:** blood glucose monitoring devices, cost saving, electronic decision support tool, irrational prescribing, prescribing guidelines, prescription compliance

## Abstract

Introduction

The Saudi Ministry of Health implemented prescribing guidelines and an integrated Decision Support Tool within the electronic prescription system to enforce eligibility, quantity, and timing criteria for blood glucose monitoring devices. This study assessed guideline compliance, estimated potential cost savings, and modeled the potential expansion in patient coverage resulting from the rejection of non-compliant prescriptions.

Methods

This was a cross-sectional, descriptive study. Data were collected from the records of the electronic prescription system ("Wasfaty"), covering prescriptions for blood glucose monitoring devices issued between January and December 2023, both overall and by specific item. The data also included the number of prescriptions rejected due to non-compliance with guidelines, both in total and by item type, and the costs of the different blood glucose monitoring devices. Potential new patient coverage was modeled by dividing rejected items by the standard per-patient allocation over the prescribed interval.

Results

The Decision Support Tool rejected 1,600,517 non-compliant prescriptions for blood glucose monitoring devices, constituting 0.9% of the total prescriptions issued in 2023. Continuous glucose monitoring (CGM) system readers showed the highest non-compliance rate (64.6%), followed by glucometers (49.3%) and CGM system sensors (31.1%), while glucometer strips and lancets had minimal non-compliance rates (0.70% and 0.50%, respectively). The potential cost savings from rejecting non-compliant prescriptions totaled approximately USD 25.6 million (96 million Saudi Riyals). The greatest savings came from flash glucose monitoring system sensors (62.5%), followed by glucometer devices (14.6%), and glucometer strips (11.5%). The rejection of non-compliant prescriptions could enable expanded coverage of new patients, including 63,367 annually for glucometer strips, 56,399 for glucometer lancets, 212,529 for glucometers, 64,394 for glucose monitoring system sensors, and 28,025 for glucose monitoring system readers, based on standard prescription intervals.

Conclusion

Non-compliance was highest for CGM system readers, glucometers, and CGM system sensors. The rejection of non-compliant prescriptions through prescribing guidelines using the Decision Support Tool potentially leads to cost savings and expansion of blood glucose monitoring device coverage. Further research in Saudi Arabia is needed to identify factors influencing guideline adherence and to evaluate the intervention’s impact on clinical outcomes.

## Introduction

Blood glucose monitoring enables individuals with diabetes to effectively manage their glycaemic control while also offering clinicians valuable insights into the patient’s glycaemic profile and treatment strategies [1]. Patients with diabetes can monitor their blood glucose levels using either fingerstick self-monitoring of blood glucose (SMBG) [2,3] or continuous glucose monitoring (CGM), which employs transcutaneous electrochemical systems to continuously measure glucose levels in interstitial fluid [2]. Both SMBG and CGM enable real-time detection of glycemic fluctuations, enhancing safety and supporting timely lifestyle and therapeutic adjustments [3,4].

International guidelines have been established to identify the target populations for blood glucose monitoring and the frequency of blood glucose testing. Both SMBG and CGM are recommended for individuals on intensive insulin regimens (including adults and youth with type 1 diabetes) by the American Association of Clinical Endocrinology Clinical Practice Guideline (2021) and the American Diabetes Association Standards of Medical Care in Diabetes (2020) [4,5]. The American Diabetes Association Standards of Medical Care in Diabetes (2020) also recommend SMBG for patients on non-insulin therapies who require adjustment of diet, physical activity, or medications, and CGM for individuals with type 2 diabetes who have not achieved glycemic targets [5].

Regarding the frequency of blood glucose testing, the American Diabetes Association guidelines recommend that patients using intensive insulin regimens should be encouraged to monitor glucose levels using SMBG (and/or CGM) at least once every eight hours [5]. In contrast, there are no established guidelines for testing frequency in non-insulin-treated type 2 diabetes patients. However, a meta-analysis of randomized controlled trials suggests that SMBG should be used for these individuals, with a recommended frequency of eight to 14 tests per week [6].

Determining the eligible patients and the testing frequency plays a crucial role in shaping the pattern of blood glucose monitoring (BGM) device usage. Studies examining these trends have revealed varied patterns over recent decades. Some research has reported a rise in glucose test strip utilization, reflected in both the increasing number of users and the average number of test strips dispensed [7,8], while other studies have noted stable usage patterns [9]. However, more critical than these trends is the rational and appropriate prescription of BGM devices for patients with clear clinical indications.

Studies from various countries have reported the irrational prescribing of BGM devices, often involving their distribution to ineligible patients [10-12]. Routine self-monitoring of blood glucose in non-eligible patients, such as those with diabetes not treated with insulin, may lead to increased anxiety and depression scores [13,14] and is associated with resource wastage [7,10]. These concerns have prompted the development of clinical practice guidelines recommending appropriate use and testing frequency [15].

In Saudi Arabia, the prevalence of diabetes among adults aged 20-79 years reached 17.7% in 2022, while the incidence rate of type 1 diabetes among children aged 0-14 years was 31.4 per 100,000 population per year, placing the country among the top 10 globally for this indicator [16]. The Saudi Diabetes Clinical Practice Guidelines recommend CGM for children and adults with type 1 diabetes, and SMBG for patients with both type 1 and type 2 diabetes [17]. BGM devices are publicly funded for eligible groups and prescribed by physicians in public healthcare facilities through the Electronic Prescription System "Wasfaty", enabling patients to access them free of charge.

To reduce the risk of overuse, avoid unnecessary costs, and ensure equitable access to BGM devices, the Ministry of Health implemented a comprehensive intervention. This intervention includes prescribing guidelines that define patient eligibility criteria, indications for BGM devices, the allowable quantity of items per prescription, and the minimum interval required between prescriptions. The guidelines cover the prescription of both flash glucose monitors and glucometers. Eligibility criteria for flash glucose monitors include a confirmed diagnosis of type 1 diabetes and an age above four years. The quantity limits are one reader and six sensors per prescription, with refill intervals of at least 1,800 days for readers and 90 days for sensors. For glucometers, lancets, and test strips, eligibility requires a confirmed diagnosis of type 1 or type 2 diabetes, with limits of one glucometer, up to 200 lancets, and two packs of 200 test strips per prescription. Refill intervals are set at a minimum of 1,000 days for glucometers and 60 days for lancets and test strips from the last dispensed items. To support adherence to these guidelines, an electronic governance measure was introduced in the form of a point-of-care Electronic Decision Support Tool (EDST). The EDST is integrated into the "Wasfaty" electronic prescription system and linked to each patient’s medical record. It automatically rejects prescriptions that do not meet eligibility criteria, exceed the recommended quantity, or are requested at inappropriate intervals.

Evaluating the impact of this intervention is critical for informing decisions regarding its continuation and potential scalability across other areas of healthcare delivery. This study aimed to assess compliance with national prescribing guidelines for BGM devices and to estimate the potential cost savings resulting from the rejection of non-compliant prescriptions through the EDST. In addition, it aimed to model the potential one-year increase in BGM device coverage achievable through the redistribution of devices saved by the EDST.

## Materials and methods

Prescription guidelines and EDST

The "Wasfaty" prescription system is a computerized platform that enables physicians to issue prescriptions electronically by selecting the health facility, initiating a prescription, identifying the patient, recording the diagnosis, and adding the required items. Within this system, the EDST evaluates each prescription by automatically checking eligibility criteria, quantity limits, and refill intervals against linked patient records. Prescriptions that meet all requirements are accepted, whereas any prescription that violates eligibility, quantity, or duration rules is automatically rejected and cannot be dispensed to the patients. Alerts for each rejected prescription are recorded in the Wasfaty system. Repeated non-compliant prescription attempts for the same patient are counted as separate rejections.

Study design, variables, and source of data

This was a descriptive, cross-sectional, nationwide study covering all Ministry of Health facilities from all regions of the country. We used the 2023 database of the Electronic Prescription System "Wasfaty," which captures all medications, equipment, and supplies prescribed at public health facilities and dispensed at the retail pharmacies to individuals eligible for public drug coverage. In this database, we targeted the individual items of BGM devices that were prescribed between January and December 2023. These items included glucometer devices, glucometer test strips, glucometer lancets, flash glucose monitoring system sensors, and flash glucose monitoring system readers. We collected data on the number of prescriptions for each item that were accepted and dispensed and the number of prescriptions for each item that were rejected due to non-compliance with prescription guidelines. Additionally, we obtained data on the direct cost of each BGM device item from the General Directorate of the Specialized Centers at the Ministry of Health.

Data analysis

Descriptive statistical methods were employed to summarize the data. The total number of rejected items for each type of BGM device, as well as the total number of rejected items across all device types, was reported. The relative contribution of each category to the total number of rejected prescriptions was expressed as a percentage.

Cost data were analyzed using descriptive statistics to estimate the total potential cost savings resulting from the rejection of ineligible prescriptions. Cost savings for each item type were determined by multiplying the number of rejected prescriptions by the corresponding unit cost. The total cost was calculated by summing the estimated cost for all BGM device items. The percentage contribution of each device type to the total estimated cost savings was then computed.

The potential increase in patient coverage was estimated for a 12-month period by modelling the number of patients that could be served according to standard prescription rules of the Saudi Diabetes Clinical Practice Guidelines [17]. A potential increase in patient coverage was modeled by dividing the total number of saved blood glucose monitoring devices by the number of devices normally prescribed per patient over 12 months. Exceptions were applied for glucometers, prescribed at a rate of one device every 1,000 days, and for CGM readers, prescribed once every 1,800 days; for these device types, coverage was modelled as one device per patient per year. All analyses were conducted using Microsoft Excel (Microsoft Corp., Redmond, WA, USA).

The study proposal was approved by the Institutional Review Board of the Saudi Ministry of Health (IRB log number: 25-33M; dated March 12, 2025).

## Results

During 2023, a total of 177,159,391 prescriptions for BGM devices were attempted in public health facilities (Table 1). Of these, 1,600,517 prescriptions (0.90%) were deemed ineligible and rejected by the EDST. Approximately 289,000 rejected prescriptions were for flash CGM devices prescribed to patients with type 1 diabetes, while 1,311,339 were for glucometers prescribed to patients with both type 1 and type 2 diabetes (Table 1).

**Table 1:** Prescriptions for blood glucose monitoring devices not complying with prescription guidelines and rejected by the Electronic Decision Support Tool in 2023 (N=177,159,391)

Item	Total prescribed	Number rejected	Percent rejected
Glucometer strips	107,880,263	760,413	0.70
Glucometer lancets	67,963,997	338,397	0.50
Glucometer device	431,404	212,529	49.26
Glucose monitoring system sensor	840,352.00	261,153	31.08
Glucose monitoring system reader	43,375.00	28,025	64.61
Total	177,159,391	1,600,517	0.90

Among rejected items, glucose monitoring system readers had the highest rejection rate, with nearly two-thirds of prescriptions rejected (64.61%; 28,025 of 43,375), followed by glucometer devices, with approximately half rejected (49.26%; 212,529 of 431,404). Glucose monitoring system sensors were rejected in about one-third of prescriptions (31.1%; 261,153 of 840,352). Glucometer strips and lancets had the lowest rejection rates, at 0.70% and 0.50%, respectively (Table 1).

**Figure 1:**
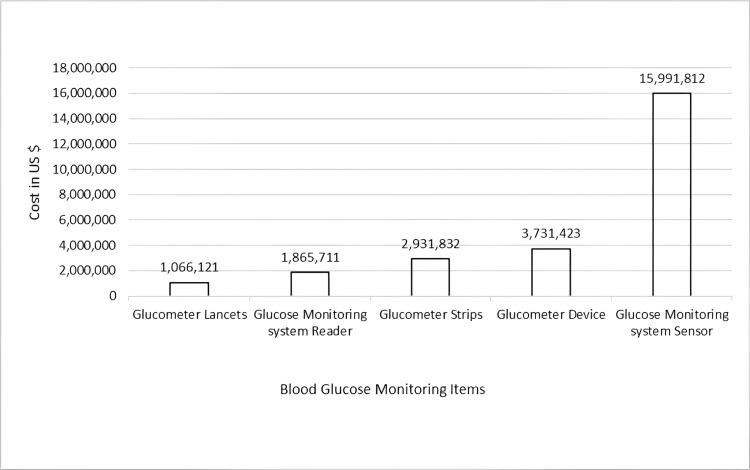
Potential cost savings (USD) from rejected non-compliant prescriptions by the Electronic Decision Support Tool in 2023 USD: US dollars

The potential cost savings resulting from the rejection of prescriptions not complying with guidelines totaled approximately USD 25.6 million (96 million Saudi Riyals) in 2023 (Figure 1). The largest share (62.5%) was attributed to flash glucose monitoring system sensors (USD 16.0 million; 60 million Saudi Riyals ), followed by glucometer devices with a share of 14.6% (USD 3.7 million; 14 million Saudi Riyals) and glucometer strips with a share of 11.5% (USD 2.9 million; 11 million Saudi Riyals ) (Figure 1). The lowest savings were observed for glucometer lancets, which accounted for USD 1.1 million (4.2%) of the total (Figure 1).

The rejection of prescriptions, along with the corresponding eligibility rules, was used to model the potential increase in coverage of new patients with BGM devices (Table 2). Based on standard prescription guidelines, glucometer lancets, prescribed as two 200-unit packets every 60 days, and glucometer strips, prescribed as 200-unit packets every 60 days, could support approximately 63,367 and 56,399 new patients annually, respectively. Glucometer devices, with a usage interval of one device per 1,000 days, could potentially cover 212,529 new patients. Glucose monitoring system sensors, prescribed as six sensors every 90 days, could serve 64,394 patients per year, while glucose monitoring system readers, intended for use once every 1,800 days, could support 28,025 patients annually.

**Table 2:** Potential increase in coverage by the saved blood glucose monitoring devices' items

Item	Quantity available	Prescription rule	Potential number of patients covered during one year
Glucometer strips	760,413	2 packets every 60 days	63,367
Glucometer lancets	338,397	1 packet every 60 days	56,399
Glucometer device	212,529	1 device every 1000 days	212,529
Glucose monitoring system sensor	261,153	6 sensors every 90 days	64,394
Glucose monitoring system reader	28,025	1 reader every 1800 days	28,025

## Discussion

The aim of this study was to evaluate compliance with national prescription guidelines for BGM devices, quantify the potential cost savings resulting from an EDST’s rejection of non-compliant prescriptions, and model the potential one-year increase in BGM device coverage achievable through the redistribution of devices saved by electronic DST. The findings show that 0.9% (≈1.6 million) of all BGM-related prescriptions issued in 2023 in public health facilities did not comply with national prescription guidelines and were consequently rejected by the electronic DST. These rejected prescriptions correspond to an estimated potential cost savings of approximately USD 25.6 million (96 million Saudi Riyals). The quantities saved, as modeled in this study, have the potential to substantially increase patient coverage across all BGM device categories.

The intervention implemented by the Saudi Ministry of Health comprised two key components. The first involved establishing eligibility criteria and limits on the quantity of BGM items allowed per prescription. Similar interventions, designed to restrict the volume of BGM supplies dispensed to patients over defined time intervals, have been employed in other healthcare systems to promote more appropriate use of blood glucose test strips (BGTS). These measures have shown substantial potential to reduce unnecessary utilization and generate significant cost savings [18]. For instance, in Ontario, Canada, the introduction of a policy aligning BGTS quantity limits with clinical guidelines resulted in a substantial and lasting change in utilization patterns, generating nearly USD 24 million (90.0 million Saudi Riyals) in savings within one year, primarily among individuals with diabetes not using insulin [18]. Likewise, in British Columbia, Canada, the introduction of a similar policy led to an immediate reduction in average BGTS use and provincial expenditures, yielding estimated savings of USD 44.6 million (167.2 million Saudi Riyals) between 2015 and 2019 [19].

In jurisdictions such as Ontario and British Columbia, Canada, as in Saudi Arabia, blood glucose monitoring devices and their supplies are publicly funded for eligible patients, thereby reducing direct out-of-pocket expenditures. However, as in other third-party payer systems, this model may contribute to the inefficient utilization of medical resources by both healthcare providers and patients [20,21]. Such patterns of overutilization can result in increased healthcare system costs and may limit the availability of essential services for other patients [20,21]. For example, a study in Italy reported that the excessive prescription of SMBG strips contributed to cost overruns and resulted in some patients, particularly those with insulin-treated diabetes, not receiving a sufficient number of strips to adequately monitor their blood glucose levels [22]. Therefore, addressing the over-prescription of glucose monitoring devices and supplies can free up valuable healthcare resources. The resulting cost savings could be redirected toward other diabetes care initiatives that may yield greater improvements in quality of life and long-term outcomes for individuals with diabetes [23].

Second, the Saudi Ministry of Health supported the implementation of BGM prescribing guidelines through the integration of an electronic EDST within the national electronic prescription system. A study evaluating similar efforts in Canada found that relying solely on the dissemination of guidelines, without supportive implementation tools, had little impact on reducing the overuse of glucose monitoring strips, largely due to the complexity of the guidelines and the absence of practical mechanisms to facilitate their application in routine clinical practice [15]. In general, educational tools, including clinical practice guidelines, have been found to be ineffective in consistently changing clinical practice behaviors. Numerous studies have shown that physicians often struggle to adhere reliably to these guidelines, commonly due to limited awareness and insufficient familiarity [24]. Clinical Decision Support Systems are health information technologies integrated into electronic health records that provide clinicians with timely, evidence-based alerts and patient-specific information to promote adherence to recommended care guidelines [25,26]. The integration of Clinical Decision Support Systems with clinical guidelines has been shown to enhance healthcare providers’ adherence to recommended care standards [24,27].

Our findings indicate that prescriptions for continuous glucose monitoring sensors were among the three most non-compliant prescriptions and were associated with the greatest potential cost savings. This could be attributed to their relatively high volume in addition to their high unit cost, which was reported as a major contributor to the overall cost of diabetes management [28]. Conversely, we observed a low proportion of glucometer test strip prescriptions that did not comply with national guidelines, warranting further research to identify factors contributing to adherence to these guidelines

A limitation of this study was that we did not measure the effect of the intervention on the clinical outcomes of patients with diabetes; however, other studies assessing these parameters have reported no detrimental effects. In British Columbia, a population-based study spanning seven years found that the introduction of BGTS quantity limits had no immediate impact on emergency department visits for hyperglycemia or hypoglycemia, nor on hemoglobin A1c levels [29]. Similarly, a study involving patients with type 2 diabetes found that changes in the frequency of strip use had no effect on glycemic control [30]. Nevertheless, further studies within the context of Saudi Arabia are needed to provide empirical evidence on the impact of this intervention on clinical outcomes. Another limitation was that the database was anonymized and lacked information on patients’ treatment types and other background characteristics, which restricted our ability to analyze study outcomes across different patient groups. Additionally, this study represents a single-year, descriptive analysis limited to public Ministry of Health facilities using the Wasfaty system; therefore, no causal inferences or temporal trends can be drawn, and the findings are not generalizable to the private healthcare sector.

## Conclusions

The implementation of blood glucose monitoring prescribing guidelines alongside a Decision Support Tool was associated with a reduction in prescriptions not complying with national guidelines, potentially leading to meaningful cost savings. These savings may allow for reallocation within the healthcare system to enhance coverage and access to essential diabetes management devices for more patients. Further research in Saudi Arabia is warranted to explore factors influencing guideline adherence and to assess the intervention’s impact on clinical outcomes.
